# Skin biology and ageing

**DOI:** 10.1002/ski2.458

**Published:** 2024-11-13

**Authors:** Abigail K. Langton, Rachel E. B. Watson

**Affiliations:** ^1^ Centre for Dermatology Research School of Biological Sciences Faculty of Biology, Medicine & Health The University of Manchester Manchester UK; ^2^ Agency for Science, Technology & Research (A*STAR) Skin Research Labs & Skin Research Institute of Singapore Singapore Republic of Singapore

The skin serves as the body's primary defence against our external environment and throughout the life course, is vulnerable to an accumulation of damage which, in the long term, impacts the ability of skin to serve this primary purpose. Maintaining healthy skin and preventing skin disease are crucial for all, but as the proportion of the population who are old (>65 years) and very old (>85 years) grows—particularly in industrialized countries—the dermatological needs of the elderly will become increasingly important. Skin ageing is a complex process influenced by both internal and external factors that gradually erode the skin's structural integrity and physiological function. Whilst intrinsic ageing is an inevitable part of life, shaped by genetics and the passage of time, external factors—many within our control—also play a significant role. From sun exposure and pollution to lifestyle habits such as smoking, dietary choices and sleep patterns, these external influences contribute additional biological stimuli which impact the rate of cutaneous ageing.

In this special edition on ‘Skin Biology and Ageing’, we present papers that highlight the diversity in basic and clinical skin health research which aims to better understand how skin health alters across the lifespan. Sun‐related behaviours and attitudes emerge as a central theme in this special edition. Ultraviolet radiation (UVR) is a complete carcinogen, both initiating tumourigenesis and promoting skin cancer, and while preventive measures such as wearing sunscreen and protective clothing can reduce skin cancer rates in individuals with lightly pigmented skin, the adoption of such behaviours remains low. In this issue, Cherfrere et al.^1^ explored whether there is a link between self‐perception of general health and sun‐related behaviours. Interestingly, those with a positive self‐perception of their health were more likely to enjoy tanning, spending time in the sun and had a history of sunburns even though they also used sunscreen. From an Old World or Western perspective, our attitudes to tanning have changed enormously over the past century; where tanned skin was once seen as a visual clue to lower socio‐economic class, the advent of cheaper travel in the post‐war era combined with the cult of celebrity have reinforced the concept that modelling a tan is a sign of affluence or prosperity. The findings presented here suggest that stronger health messaging about the dangers of excessive sun exposure and skin cancer risk may be particularly necessary for individuals who perceive themselves as healthy.

Public health campaigns promoting photoprotection and skin cancer awareness have been in place since the early 1980s and can be effective in encouraging better sun protection habits. However, despite clear evidence that regular sunscreen use reduces skin cancer risk, adolescents and young adults often neglect photoprotective behaviours. In this issue, Johnson et al.^2^ highlight the effectiveness of an educational video in enhancing sun safety practices among student athletes. This research is promising, as it shows that digital education can be widely accessible and tailored to specific groups.

Moreover, healthcare workers are a unique audience for health education due to their direct role in patient care. Porter et al.^3^ present findings from a cross‐sectional prospective observational study examining the impact of a hospital‐based digital sun protection campaign on healthcare workers' sun‐related attitudes and behaviours. Initially, the sun protection practices among those surveyed were varied—while sunscreen use was common, practices like seeking shade and wearing protective clothing or hats were less so. However, the campaign positively influenced self‐reported sun protection habits, increased skin cancer awareness and boosted healthcare workers' confidence in discussing sun safety with others. This study underscores the importance of educating healthcare professionals and empowering them to share health‐related messages with their patients and the broader community.

Excessive UVR exposure is the primary cause of premature skin ageing in individuals with lighter skin and has been strongly linked to increased facial wrinkling and wrinkle depth. Emerging evidence suggests that the epigenome plays a significant role in the skin's response to UVR. In their article, Barnes et al.^4^ review recent studies on skin ageing and carcinogenesis, highlighting disruptions across various levels of the epigenetic landscape. Additionally, Bai et al.^5^ explore the clinical potential of exosomes in regenerative and cosmetic dermatology. Exosomes, which are extracellular vesicles that carry bioactive substances during both normal and abnormal cellular processes, hold promise as biomarkers and targeted drug delivery systems. Although current clinical trials on the cosmetic use of exosomes are limited, advancements in this technology could offer exciting new possibilities for skin repair and the rejuvenation of both intrinsically aged and photodamaged skin.

The research presented in this special edition highlights the complexity of skin biology and underscores the importance of both preventive measures and innovative treatments in addressing dermatological challenges throughout life. From effective sun safety campaigns to the potential of emerging technologies like exosomes, the work featured here demonstrates the need for a comprehensive approach to skin health. With an ageing population and the rising incidence of UVR‐related conditions, it is vital that we continue to prioritize research, education and public health initiatives to support healthy skin at every stage of life.

## CONFLICT OF INTEREST STATEMENT

The authors declare no conflicts of interest.

## AUTHOR CONTRIBUTIONS

**Abigail K. Langton**: Writing—original draft (equal); writing—review & editing (equal). **Rachel E. B. Watson**: Writing—original draft (equal); writing—review & editing (equal).

## FUNDING INFORMATION

This article received no specific grant from any funding agency in the public, commercial, or not‐for‐profit sectors.

## ETHICS STATEMENT

Not applicable.

## PATIENT CONSENT

Not applicable.

## Data Availability

Data sharing is not applicable to this article as no new data were created or analyzed in this study.
